# Effects of apples and specific apple components on the cecal environment of conventional rats: role of apple pectin

**DOI:** 10.1186/1471-2180-10-13

**Published:** 2010-01-20

**Authors:** Tine R Licht, Max Hansen, Anders Bergström, Morten Poulsen, Britta N Krath, Jaroslaw Markowski, Lars O Dragsted, Andrea Wilcks

**Affiliations:** 1 Department of Microbiology and Risk Assessment, National Food Institute, Technical University of Denmark, Mørkhøj Bygade 19, DK-2860 Søborg, Denmark; 2 Department of Toxicology and Risk Assessment, National Food Institute, Technical University of Denmark, Mørkhøj Bygade 19, DK-2860 Søborg, Denmark; 3 Department of Human Nutrition, Faculty of Life Sciences, University of Copenhagen, Rolighedsvej 30, DK-1958 Frederiksberg C, Denmark; 4 Department of Storage and Processing, Research Institute of Pomology and Floriculture, 96-100 Skierniewice, Poland

## Abstract

**Background:**

Our study was part of the large European project ISAFRUIT aiming to reveal the biological explanations for the epidemiologically well-established health effects of fruits. The objective was to identify effects of apple and apple product consumption on the composition of the cecal microbial community in rats, as well as on a number of cecal parameters, which may be influenced by a changed microbiota.

**Results:**

Principal Component Analysis (PCA) of cecal microbiota profiles obtained by PCR-DGGE targeting bacterial 16S rRNA genes showed an effect of whole apples in a long-term feeding study (14 weeks), while no effects of apple juice, purée or pomace on microbial composition in cecum were observed. Administration of either 0.33 or 3.3% apple pectin in the diet resulted in considerable changes in the DGGE profiles.

A 2-fold increase in the activity of beta-glucuronidase was observed in animals fed with pectin (7% in the diet) for four weeks, as compared to control animals (P < 0.01). Additionally, the level of butyrate measured in these pectin-fed animal was more than double of the corresponding level in control animals (P < 0.01). Sequencing revealed that DGGE bands, which were suppressed in pectin-fed rats, represented Gram-negative anaerobic rods belonging to the phylum *Bacteroidetes*, whereas bands that became more prominent represented mainly Gram-positive anaerobic rods belonging to the phylum *Firmicutes*, and specific species belonging to the *Clostridium *Cluster XIVa.

Quantitative real-time PCR confirmed a lower amount of given *Bacteroidetes *species in the pectin-fed rats as well as in the apple-fed rats in the four-week study (P < 0.05). Additionally, a more than four-fold increase in the amount of *Clostridium coccoides *(belonging to Cluster XIVa), as well as of genes encoding butyryl-coenzyme A CoA transferase, which is involved in butyrate production, was detected by quantitative PCR in fecal samples from the pectin-fed animals.

**Conclusions:**

Our findings show that consumption of apple pectin (7% in the diet) increases the population of butyrate- and β-glucuronidase producing *Clostridiales*, and decreases the population of specific species within the *Bacteroidetes *group in the rat gut. Similar changes were not caused by consumption of whole apples, apple juice, purée or pomace.

## Background

While the beneficial effects of fruits and vegetables on human health are widely acknowledged due to a number of epidemiological [1] and intervention studies [2,3], the mechanisms behind such effects remain largely unknown. In the integrated European project, ISAFRUIT http://www.isafruit.org, we have set out to uncover effects of apple consumption on a number of biological parameters, as well as to reveal the underlying mechanisms causing these effects. Apples were chosen as study object, since apples are among the types of fruits consumed in highest amounts throughout the European Union.

One of the possible ways for apples and other foods to affect human health parameters is through alteration of the composition and activity of the intestinal microbiota. Recent publications have revealed effects of vegetables and fruit products on the bacterial population of the gut [4,5]. Large efforts are presently put into studies on the importance of the intestinal microbiota for health. A number of health related targets may be affected by the intestinal microbiota, including the immune system [6], targets related to cancer prevention [7], resistance to infections [8] and obesity [9]. Knowledge about the mechanisms involved in beneficial effects of apples may contribute to the design of novel prebiotic substances.

The main purpose of our study was to identify effects of consumption of apples or apple products on the microbial populations in the rat cecum. Since the cultivable part of the fecal microbiota probably constitutes only 20-50% of the gut microbes [10], it is important to explore effects on this complex ecosystem by use of molecular fingerprinting methods allowing representation of the non-cultivable bacterial species.

Denaturing Gradient Gel Electrophoresis (DGGE) of PCR-amplified 16S rRNA genes have previously proved very useful for analysis of intestinal bacteria [11-13]. In the present investigation we have used this method for analysis of cecal 16S rRNA fragments amplified with universal primers, targeting the whole bacterial community. Quantitative real-time PCR was used in order to verify changes observed by DGGE. Additionally, we studied selected cecal parameters that could be influenced by a changed microbiota. These included measurements of short-chain fatty acids (SCFA), which have potentially beneficial effects on gut health, as well as of the potentially adverse enzymes synthesized by colonic bacteria, β-glucosidase (BGL) and β-glucuronidase (GUS).

## Results

### Effect of long-term apple consumption on the rat cecal environment (Experiment A)

Consumption of 10 g apples a day for a period of 14 weeks had no effect on cecal pH, relative cecal weight, or production of SCFA (data not shown). Apple consumption led to a small increase (mean ± standard deviation) in the activity of cecal β-glucuronidase (GUS) from 5.2 ± 2.9 U/g cecal content in 32 control animals to 6.8 ± 2.9 U/g in 32 animals fed with 10 g apples per day (P < 0.05) and an increase in beta-glucosidase (BGL) from 3.5 ± 1.1 to 4.6 ± 1.6 U/g cecal content (P < 0.05). DMH treatment of 16 animals within each of the groups, ending 6 weeks before euthanization, had no effect on any of these observations.

**Figure 1:**
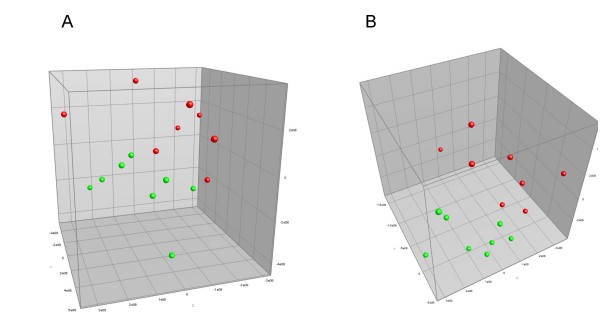
**PCA analysis of samples from Experiment A**. Principal Component Analysis of DGGE profiles of bacterial rRNA genes present in fecal samples from rat fed with control diet (green) or 10 g apples a day (red), respectively. A: Uninitiated animals. The amount of variability accounted for by Factor X is 25.0%, by Factor Y 16.2% and by Factor Z 13.6%. B: DMH initiated animals. The amount of variability accounted for by Factor X is 31.6%, by Factor Y 14.3% and by Factor Z 12.0%. Comparison of initiated and uninitiated animals by PCA revealed no grouping related to DMH initiation.

Principal Component Analysis (PCA) of DGGE profiles containing 16S ribosomal genes amplified by universal bacterial primers revealed that apple consumption affected the composition of bacteria in cecal samples (Figure 1). However, it was not possible to explain this effect by occurrence of specific bands, and thus not possible to identify specific bacterial species affected by the apple diet.

### Effect of long-term consumption of apple purée, pomace, pectin, and juice on the rat cecal environment (Experiment B)

To clarify which of the components present in apples that caused the increase in enzymatic activity as well as the changes in cecal bacterial composition, a number of different apple components were tested for 14 weeks in seven groups of 16 initiated animals as described in Materials and Methods. No effect was observed of any of the components tested on cecal pH, cecal weight and GUS activity of the rats (Table 1). The level of cecal BGL activity was lower in the group fed whole apple purée compared to all other groups, including the control group (P < 0.05). None of the components had any effect on the cecal concentrations of acetate and propionate. In the pomace and the 3.3% pectin groups, there were significant increases in the concentration of butyrate from 14.3 ± 3.7 μmol/g cecal content in the control group to 27.9 ± 12.6 μmol/g in the rats fed pomace (P < 0.01) and 20.8 ± 11.8 μmol/g in the rats fed pectin (P < 0.05) (Table 1).

**Table 1:** Cecal parameters from Experiment B

Dietary group	Control	Puree	Cloudy juice	0.33% pectin	Clear juice	Pomace	3.3% pectin
Acetate (μmol/g cecal content)	98.0 ± 26.6	94.7 ± 30.0	81.5 ± 40.0	86.7 ± 39.7	79.3 ± 27.8	110.9 ± 29.9	101.9 ± 36.7
Propionate (μmol/g cecal content)	25.7 ± 5.8	24.9 ± 7.4	22.2 ± 10.1	22.6 ± 10.0	21.7 ± 8.7	27.2 ± 8.0	22.8 ± 6.0
Butyrate (μmol/g cecal content)	14.3 ± 3.7	16.5 ± 7.2	15.7 ± 10.4	15.2 ± 12.5	15.2 ± 8.0	27.9 ± 12.6**	20.8 ± 11.8*
Cecal pH	7.1 ± 0.1	7.1 ± 0.1	7.1 ± 0.3	7.2 ± 0.3	7.2 ± 0.1	7.0 ± 0.1	7.2 ± 0.4
Relative cecum weight (g/kg b.w.)	7.4 ± 1.5	9.2 ± 1.8	8.0 ± 1.3	7.9 ± 1.5	8.7 ± 2.0	8.8 ± 1.7	8.9 ± 2.2
GUS (U/g cecal content)	5.9 ± 1.8	6.4 ± 2.4	7.2 ± 3.2	7.2 ± 3.4	6.5 ± 3.4	6.5 ± 2.5	7.6 ± 2.7
BGL (U/g cecal content)	5.2 ± 2.2	3.9 ± 1.1**	5.0 ± 2.6	5.9 ± 2.5	4.4 ± 1.1	5.3 ± 1.2	5.7 ± 1.9

The data are averages and standard deviations from 16 animals in each group.

* Asterisks indicate a significant difference from the control group; P < 0.05 (*) or P < 0.01 (**). U is defined as μmol/h.

**Figure 2:**
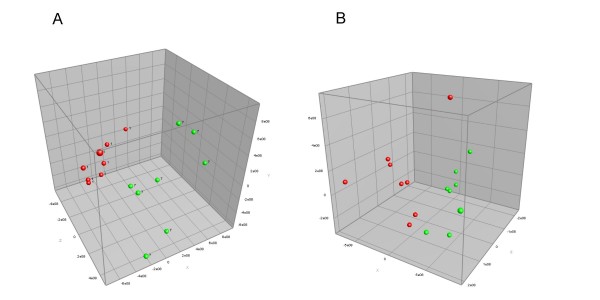
**PCA analysis of samples from Experiment B**. Principal Component Analysis of DGGE profiles of bacterial rRNA genes present in fecal samples from rat fed with control diet (red) or pectin diet (green), respectively. A: Pectin in diet constituted 3.3%. The amount of variability accounted for by Factor X is 25.5%, by Factor Y 19.6% and by Factor Z 13.8%. B: Pectin in diet constituted 0.33%. The amount of variability accounted for by Factor X is 36.4%, by Factor Y 22.1%, and by Factor Z 10.7%.

Comparison of DGGE profiles containing 16S ribosomal genes amplified from cecal contents by universal bacterial primers showed no convincing difference between the control animals and animals fed with either apple purée, pomace, clear or cloudy juice (data not shown). However, animals fed with either 0.33% or 3.3% pectin had a clear difference in their composition of cecal bacteria, which was illustrated by PCA (Figure 2).

### Effect of short-term consumption of apple and apple pectin on the rat cecal environment (Experiment C)

To further elucidate the observed effects of whole apples and apple pectin, three groups of eight rats were fed with either control diet, 10 g apples a day or 7% pectin for a period of four weeks. There was no significant effect on cecal BGL activity of the rats, but a significant (P < 0.01) increase in the activity of GUS was observed from 4.1 ± 1.2 U/g cecal content in control animals to 10.7 ± 5.6 U/g in animals fed with pectin (Table 2). In animals fed 7% pectin there was an increase (P < 0.01) in the production of cecal butyrate, a decrease in cecal pH (P < 0.01) and an increase in cecal weight relative to total animal weight (P < 0.01). The apple fed rats also had a significant drop in cecal pH (P < 0.05) and increase in butyrate (P < 0.05), but no changes in GUS or cecal weight (Table 2).

**Table 2:** Cecal parameters from experiment C.

Dietary group	Control	7% pectin	10 g apple
Propionate (μmol/g cecal content)	6.8 ± 2.3	10.5 ± 4.4	10.2 ± 4.1
Butyrate (μmol/g cecal content)	3.7 ± 2.2	9.4 ± 3.1**	6.7 ± 4.5*
Cecal pH	7.0 ± 0.1	6.6 ± 0.2**	6.8 ± 0.3*
Relative cecum weight (g/kg b.w.)	12.3 ± 1.9	19.0 ± 5.2**	15.2 ± 5.4
GUS (U/g cecal content)	4.1 ± 1.2	10.7 ± 5.6**	5.9 ± 2.9
BGL (U/g cecal content)	3.5 ± 0.6	4.9 ± 1.8	3.8 ± 1.8

The data are averages and standard deviations from eight animals in each group.

* Asterisks indicate a significant difference from the control group; P < 0.05 (*) or P < 0.01 (**). U is defined as μmol/h.

**Figure 3:**
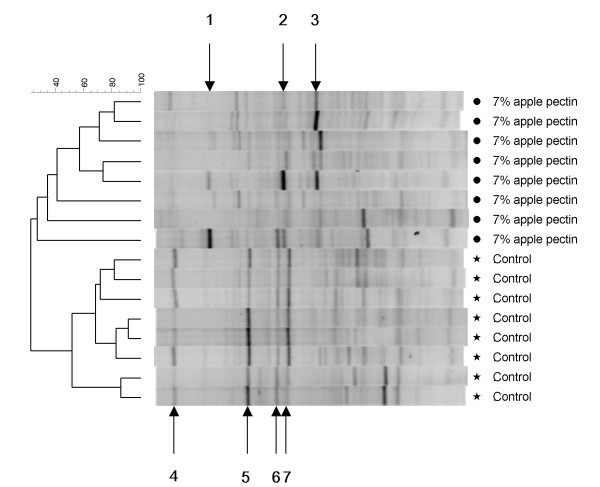
**Cluster analysis of samples from Experiment C**. Pearson correlation analysis of universal DGGE gel profiles from cecal content of rats fed with either control- or 7% pectin diet for four weeks. Bands indicated by arrows represents Anaeroplasma (1), Clostridium sp. (2), Clostridiales (3), Bacteroides sp. (4, 6 and 7), and Alistipes (5). Metric scale indicates degree of similarity in percent.

In the short-term experiment, PCA of the universal DGGE profiles did not reveal an effect of apple consumption (data not shown), as was observed in the long-term trial (Experiment A). However, a marked effect of pectin consumption was observed (Figure 3). Sequencing of bands, which were present on the profiles from pectin-fed animals, but not on the control profiles revealed that these bands represented species belonging to the Gram-negative genus of *Anaeroplasma*, and the Gram-positive genera *Anaerostipes *and *Roseburia*, respectively. Similarly, it was found that bands present on the control profiles but absent on the profiles from pectin-fed rats represented Gram-negative *Alistipes *and *Parabacteroides *sp (Figure 3, Table 3).

**Table 3:** Sequenced bands from Experiment C, and their closest neighbour in the RDP and GenBank databases (June 2008).

Band no.	Fragment size/bp	Phylum	Genus	Species	GenBank Acc. no.	Identity (%)
1	172	*Tenericutes*	*Anaeroplasma*	*An. bactoclasticum*	M25049	93
2	168	*Firmicutes*	*Anaerostipes*	Uncultured bacterium	AJ418974	99
3	168	*Firmicutes*	*Roseburia*	Uncultured bacterium	AY975500	99
4	187	*Bacteroidetes*	*Parabacteroides*	*Bacteroides *sp.	AF157056	100
5	179	*Bacteroidetes*	*Alistipes*	Al. massiliensis	AY547271	96
6	186	*Bacteroidetes*	*Alistipes*	Uncultured bacterium	AJ419011	99
7	194	*Bacteroidetes*	*Parabacteroides*	Uncultured bacterium	AJ812165	98

**Figure 4:**
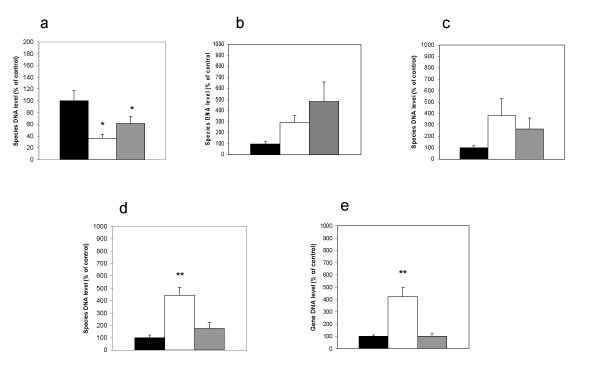
**Quantitative PCR of samples from Experiment C**. Relative amount of target gene in samples from animals in the control group (black), the pectin-fed group (white) and the apple-fed group (gray). Target genes encoded either 16S rRNA from *Bacteroides *spp. (a), *Lactobacillus *(b), *Bifidobacterium *(c), *Clostridium coccoides *(d) or the butyryl-coenzyme A CoA transferase. DNA amount in the control group was set to 100%. Error bars represent standard errors of the means. Asterisks indicate a significant difference from the control group; P < 0.05 (*) or P < 0.01 (**).

Quantitative real-time PCR was performed to verify the changes found by DGGE. *Bacteroides *16S rRNA gene content was significantly lower in both the pectin-fed group (P = 0.03) and the apple-fed group (P = 0.05) than in the control group (Figure 4a). With control levels indexed at 100%, levels were 36.6 ± 17.8% and 61.4 ± 20.0% for the pectin and apple groups, respectively.

There was no statistical significant difference in *Lactobacillus *16S rRNA gene content between the three groups (P = 0.07), however there was a trend that more lactobacilli were present in the apple-fed group (Figure 4b). Likewise, there was no significant difference in *Bifidobacterium *16S rRNA gene content between the three groups (P = 0.15), but a clear trend indicated more Bifidobacteria in the pectin-fed group than in the control group (Figure 4c).

*Clostridium coccoides *16S rRNA gene contents were significantly higher in the pectin-fed group (P < 0.001) than measured in the control group and in the apple-fed group (Figure 4d). Contents of *C. coccoides *rRNA genes in the pectin-fed rats relative to the control rats were 443.7 ± 14.8%.

Finally, the amount of the butyryl-coenzyme A CoA gene, involved in butyrate production, was significantly higher in the pectin group (P < 0.0001) than in the control group and the apple-fed group (Figure 4e). Levels relative to control were 420 ± 18.6% for the pectin group.

## Discussion

This study showed for the first time that intake of whole apples affect the microbial population in the cecum of rats. Effects were observed on the composition of the microbiota after 4 weeks as well as after 14 weeks. In the long-term feeding study the changes could be identified by PCA of the gel patterns produced by DGGE of PCR amplified 16S rRNA genes. In the short-term study, PCA did not reveal any major changes, however a statistically significant decrease in the *Bacteroides *group was observed by qPCR. This indicates that even though short-term consumption introduced minor changes in the intestinal microbiota, long-term consumption was required for these changes to be substantial enough to be detected by the PCA. The observation that long-term consumption of whole apples influenced the rat intestinal microbiota (Figure 1) is consistent with previous studies showing effects of extraction juices, rich in dietary fibers from apples, on gut microbes in rats [5,14]. In contrast to the extraction juices investigated by Sembries and coworkers, the clear and cloudy apple juices applied in the present study contained only very low amounts of dietary fibers and had no effect on the gut microbiota detectable by the methods applied.

Addition of either 0.3, 3.3 or 7.0% of dry apple pectin to the diet caused overall changes in DGGE profiles of the cecal microbiota, which for the 7% pectin group was shown to include an increase in species belonging to the Gram-negative genus of *Anaeroplasma*, and the Gram-positive genera *Anaerostipes *and *Roseburia*, and a decrease in Gram-negative *Alistipes *and *Bacteroides *spp (Figure 2 and Figure 3). Previous studies have demonstrated the ability of some *Bacteroides *species to ferment pectin [15,16] and shown an increase in the *Bacteroides *population after feeding rats with pectin related products [17]. *In vitro *fermentation studies have showed an increase in *Bacteroides *when low methylated pectin was used [18], but other fermentation studies failed to show any effect on this group [18,19]. The discrepancies between the studies may be due to differences in pectin used and/or the fact that different *Bacteroides *populations were studied.

Quantitative real-time PCR (Figure 4a) using a primer set constructed based on the sequenced bands from the DGGE analysis (Figure 3) specified that three-fold less *Bacteroides *spp were present in samples from pectin-fed rats than in the control. Additionally, a more than four-fold increase in *Clostridium coccoides*, (corresponding to the *Clostridium *cluster XIVa) in the pectin-fed animals was showed (Figure 4d). Furthermore, samples from the pectin-fed animals contained four times as many genes encoding the butyryl-coenzyme A CoA transferase as the control samples (Figure 4e). This enzyme is known to be present in bacteria from the *Clostridium *Cluster XIVa, in strains in the *Roseburia-Eubacterium rectale *cluster, and in *Faecalibacterium prausnitzii*, which are known to be numerically important butyrate-producers in the human gut [20,21].

One previous report showed a decrease in butyrate after feeding rats diet containing 5% pectin [22], however other studies have shown an increase in butyrate concentration after incubation of fecal slurry with apple pectin [23], or feeding apple pectin to weaning pigs [24]. In consistence with the observed increase in the *Clostridum *cluster XIva, as well as with another previous report [25], our study revealed a significantly higher amount of butyrate in the animals fed diet containing either 3.3% or 7% pectin (Table 1 and Table 2). Butyrate is considered to be particularly beneficial to the gut mucosa because it induces apoptosis in cancer cell lines and functions as fuel for the enterocytes [26,27].

Our results strongly suggest that the observed changes in the microbiota of the apple-fed rats should be attributed mainly to the pectin present in the apples. This is not surprising, since pectin is probably the component of the whole apple most likely to escape digestion and reach the cecal environment. However, it should be noted that the content of pectin in the apples corresponds to only approximately 0.15% in the diet, and we find it likely that also other components present in the apples contribute in concert to the observed effect on the microbiota. In support of this, it has been reported that apple pectin and a polyphenol-rich apple concentrate had more effect on cecal fermentations and lipid metabolism in rats when fed together than when fed separately [25].

In the present study, we found a significant increase in GUS enzyme activity in cecum of the 7% pectin-fed rats. This is surprising, since it contradicts a number of other reports showing that dietary pectin reduces GUS activity in the intestinal environment [28-32]. However, in consistence with our observations, Rowland and coworkers [33] reported a significant increase of GUS activity in rats after consumption of a diet containing 5% pectin, and Bauer and coworkers [34] reported a pectin-induced 10-fold increase in fecal GUS activity in pectin-fed rats. Additionally, Dabek et al. [35] reported that GUS activity is preferentially found in members of the *Firmicutes *phylum, whose populations were increased in the 7% pectin fed rats. GUS is generally considered as a biomarker for colon cancer development, since it has the potential to activate liver glucuronated toxins and mutagens [36]. However, GUS may in this way also activate beneficial compounds, such as liver glucuronated plant polyphenols [37]. Thus, the interaction between dietary pectin, GUS activity and colon carcinogenesis remains to be clarified.

## Conclusions

The reduction of pH, potentially caused by the increased SCFA production, and the increased cecal weight observed in the pectin-fed rats (7% in the diet) indicate increased cecal fermentation, which is considered beneficial for gut health. The observed increase in butyrate, which is considered beneficial for gut health, correlated with an increase in the *Clostridia *XVIa cluster that harbors many butyrate producing species. The evidence thus suggests that apples have a health-promoting effect on the rat intestinal microbiota, and that this effect is mainly explained by the presence of pectin in the apples. However, there are lots of cautions to be taken when extrapolating data from animal experiments to humans, and it should be kept in mind that rats metabolize the ingested apple components differently from humans. The data presented here will at a later stage be interpreted in the context of other biological changes recorded during the course of the ISAFRUIT project, which includes also human intervention studies.

## Methods

### Animals and housing

Male Fischer 344 rats (5-8 weeks old) were obtained from Charles River (Sulzfeld, Germany). The animals were housed two by two in standard cages. During the study the temperature was maintained at 22 ± 1°C and relative humidity at 55 ± 5%, air was changed 8-10 times per hour, and light was on from 9.00 to 21.00. Diets and acidified water (adjusted to pH 3.05 by citric acid to prevent growth of microorganisms) were provided *ad libitum*. During dosing with 1,2-dimethylhydrazine dihydrochloride (DMH) and 1 week thereafter, the animals were kept in flexible film isolators (Isotec 12134, Olac, Oxford, UK). Animal experiments were carried out under the supervision of the Danish National Agency for Protection of Experimental Animals.

### Apple products

The apples and apple products (Shampion cv. supplied by Institute for Pomology, Skierniewice, Poland) used in this study were standardized and all originated from the same harvest. Whole apples were cut in slices and the seeds were removed before serving to the rats. The exact contents of soluble solids and pectin in each of the products were known (Table 4). Obipektin A.G., Bischofszell, Switzerland, kindly provided the apple pectin.

**Table 4:** Content of soluble solids and pectin in the different apple fractions

Material	Soluble solids (%)	Unit	Total pectin	Water-soluble pectin
Whole Fruit	12.8	g/kg	4.551	0.932
Apple purée	14.5	g/kg	4.707	2.626
Cloudy apple juice	13.0	g/l	0.379	0.379
Clear apple juice	13.5	g/l	*	*
Pomace dried	-	g/kg	64.9	25.7

*Pectic substances are removed during clarification and ultrafiltration

### Diets and experimental design

#### Experiment A

64 rats were randomized (by bodyweight) in four groups of sixteen animals. After one week (Week 1) of adaptation to a control diet, two groups of animals were fed the same control diet, while two other groups were fed the control diet added 10 g raw whole apple for a period of 14 weeks until euthanization.

During Week 4-7, one of the control diet-fed groups and one of the apple-fed groups received by gavage 20 mg/kg bodyweight of DMH once a week (4 doses in total).

#### Experiment B

112 rats were randomized (by bodyweight) in seven groups of sixteen animals. After one week (Week 1) of adaptation to a control diet, the rats were fed either (i) control diet, or control diet added (ii) 10 g apple purée per day, (iii) 8 ml of cloudy apple juice per day, (iv) 8 ml of clear apple juice per day, (v) 0.5 g pomace per day, (vi) 0.33% apple pectin per day or (vii) 3.3% apple pectin per day for a period of 14 weeks until euthanization.

During Week 4-7 all animals received by gavage 20 mg/kg bodyweight of DMH once a week (4 doses in total).

#### Experiment C

24 rats were randomized (by bodyweight) in three groups of eight animals. After twelve days of adaptation to a control diet, the rats were fed either (i) control diet, (ii) control diet added 10 g apple per day, or (ii) control diet added 7% apple pectin for a period of four weeks until euthanization.

Depending on the kind of apple products, the diets were composed to ensure that all animals received the same amount of macro- and micronutrients (Table 5).

**Table 5:** Composition of the experimental diets

Ingredients (g/kg feed)	Control	Whole raw apple (10 g/rat/day)^c^	Apple puree (10 g/day/rat)^c^	Apple juice (8 ml/rat/day)^c^	Apple pomace (0.5 g/rat/day)	Pectin low (0.33%)	Pectin medium (3.3%)	Pectin high (7%)
Apple pomace	0	0	0	0	35	0	0	0
Apple pectin	0	0	0	0	0	3.3	33	70
Na-caseinate	200	232	232	232	200	200	200	200
Sucrose	100	60	0	0	100	100	100	100
Cornstarch	456	465	525	497	421	453	423	386
Soybean oil	70	80	80	80	70	70	70	70
Corn oil	80	92	92	92	80	80	80	80
Cellulose	50	22	22	50	50	50	50	50
Mineral mixture^a^	32	37	37	37	32	32	32	32
Vitamin mixture^b^	12	12	12	12	12	12	12	12

a: Containing in mg/kg diet: 2500 Ca; 1600 P; 3600 K; 300 S; 2500 Na; 1500 Cl; 600 Mg; 34 Fe; 30 Zn; 10 Mn; 0.20 I; 0.15 Mo; 0.15 Se; 2.5 Si; 1.0 Cr; 1.0 F; 0.5 Ni; 0.5 B; 0.1 B; 0.1 V; 0.07 Co.

b: Containing in mg/kg diet: 5000 (IU) vitamin A; 1000 (IU) vitamin D_3_; 50 (IU) vitamin E; 5 thiamin; 6 riboflavin; 8 pyridoxol; 2 folic acid; 0.3 D-biotin; 0.03 vitamin B-12; 20 pantothenate; 2600 cholinhydrogentartrat; 400 inositol; 40 nicotinic acid; 1 phylloquinone; 40 p-aminobenzoic acid; 1000 methionine; 2000 L-cystine.

c: Amount which is given in addition to the diet

### Sampling

Samples of cecal contents were taken from the rats directly after euthanization, and analyzed as described below. In Experiment A and B, DGGE profiling of cecal contents was performed on one animal from each cage, in Experiment C samples from all animals were analyzed.

A number of other samples were taken to analyze DMH-induced preneoplastic lesions and other biomarkers related to cancer development. However, the data obtained from these samples are not reported in the present context.

### Analysis of pH and short chain fatty acid (SCFA) composition in cecal samples

Measuring of pH was done directly in the cecal content by use of a pH-meter. Acetate, propionate, and butyrate in cecal contents were analyzed using capillary electrophoresis and indirect UV detection by a method modified from Westergaard et al. [38]. Briefly, approximately 0.1 g of cecal contents was diluted 10 times in alkaline buffer (0.1 M Tris, pH 8.7 with 100 μM malonic acid as internal standard), vortexed for 10 s, centrifuged (14000 g, 10 min, 4°C) and the supernatant was filtrated using a sterile 0.2 μm filter (Minisart). Samples were kept at -80°C until analysis. Prior to analysis the samples were diluted 30 times by running buffer (0.2 mM 1,2,4-benzenetricarboxylic acid), 8 mM TRIS and 0.3 mM tetradecyltrimethylammonium bromide, pH 7.6). The fused silica capillary (0.75 μm, 80.5 cm and 72 cm to detector window) purchased from Agilent (Waldbronn, Germany) was rinsed with 1 M NaOH before each sequence and pre-treated with water for 0.5 min, 0.1 M NaOH for 1 min and runningbuffer for 5 min before each run. Samples were injected by pressure (35 mbar, 2 s) and run at -30 kV for 12 min on a G1600A 3D Capillary electrophoresis Instrument (Hewlett-Packard, Waldbronn, Germany). All chemicals were purchased from Sigma Aldrich, Steinheim, Germany.

### Analysis of β-glucosidase (BGL) and β-glucuronidase (GUS) in cecal samples

Samples of cecal content (0.2 g) were homogenized in 1 ml phosphate buffered saline (PBS), 0.1% sodium-azide pH 7.4, and centrifuged (10000 g, 10 min, 4°C). The supernatant was used to determine the activity of BGLand GUS at 37°C on an Automated Roche/Hitachi 912 Analyzer (Roche Diagnostic GmbH, Mannheim, Germany).

BGL was measured by determining the rate of hydrolysis of the substrate p-nitrophenyl-β-D-glucopyranoside. The amount of p-nitrophenol released was measured at 415 nm with p-nitrophenol as standard. One unit (U) of enzyme was defined as the amount of enzyme that releases 1 μmol of p-nitrophenol per h. GUS was assayed by determining the rate of release of phenolphthalein from phenolphthalein-β-D-glucuronide at 540 nm with phenolphthalein as standard. One unit (U) of enzyme was defined as the amount of enzyme that releases 1 μmol of phenolphthalein from the substrate phenolphthalein-β-D-glucuronide, per hour. The specific activity for both enzymes was reported as U/g cecum content.

### Extraction of bacterial DNA from cecal samples

For DNA extraction, cecal samples were diluted 1:10 (w/vol) in PBS. DNA was extracted from 2 ml of the 10^-1 ^dilution using the QIAamp DNA Stool Mini Kit (Qiagen, Hilden, Germany) with a bead-beater step in advance, as described previously [39], and stored in 30 μl autoclaved water at -20°C until use.

### PCR amplification for DGGE

Aliquots (10 μl) of purified DNA were applied to the following to give a 50 μl PCR reaction mixture: 20 μl of 5 PRIME MasterMix (2.5×) (VWR & Bie & Berntsen, Herlev, Denmark) and 40 pmol of each of the primers. Primers HDA1-GC/HDA2 [40] targeting 16S rRNA genes from all bacteria were used in a touchdown PCR. Initial denaturation was at 96°C for 5 min, amplification was carried out using 20 cycles including denaturation at 94°C for 1 min, annealing at 65°C for 1 min decreased by 0.5°C for each cycle, and extension at 72°C for 1 min. This was followed by additional 5 cycles of denaturation at 94°C for 1 min, annealing at 55°C for 1 min, extension at 72°C for 1 min, and a final extension at 72°C for 5 min.

All PCR reactions were run on a PTC-240 DNA Engine Tetrad 2 Cycler (MJ Research, Bio-Rad Laboratories, Copenhagen, Denmark) and the products were verified by gel electrophoresis before proceeding to DGGE analysis.

### Analysis of cecal microbiota by denaturing gradient gel electrophoresis (DGGE)

DGGE was carried out as previously described [41] using a DCodeTM Universal Mutation Detection System instrument and gradient former model 475 according to the manufacturer's instructions (Bio-Rad Labs, Hercules, California). The denaturing gradient was formed with two 9% acrylamide (acrylamide-bis 37.5:1) stock solutions (Bio-Rad) in 1 × TAE (20 mM Tris, 10 mM acetate, 0.5 M EDTA, pH 7.4). The gels were made with denaturing gradients ranging from 25 to 65% for analysis of the amplified 16S rRNA fragments. The 100% denaturant solution contained 40% formamide and 7 M urea. PCR product (13 μl) were mixed with 3 μl loading dye before loading. Gels were run in 1 × TAE at 60°C for 16 hr at 36 V, 28 mA, stained with ethidium bromide for 15 min, destained for 20 min, and viewed by UV-B trans illumination at 302 nm. The BioNumerics software, version 4.60 (Applied Maths, Sint-Martens-Latem, Belgium) was used for identification of bands and normalization of band patterns from DGGE gels. Pearson correlation and Principal Component Analysis (PCA) based on DGGE pattern profiles were performed using the same software. Subtraction of averages over the characters was included in the PCA analysis.

### Excision, cloning and sequencing of selected bands from DGGE gels

Bands of specific interest were excised from DGGE gels with a sterile razor, placed in 40 μl sterile water, and incubated at 4°C for diffusion of DNA into the water. 33 μl of the sterile water (containing the DNA) was treated with S1 nuclease [42]. For sequencing of bands retrieved from universal DGGE gels, the S1 nuclease treated DNA was used in a PCR with HDA1/2 primers without GC-clamp (4 min at 94°C, 20 cycles consisting of 30 s at 94°C, 30 s at 56°C, and 1 min at 68°C, and finally 7 min at 68°C). Subsequently the PCR products were directly cloned into pCR^®^4-TOPO (Invitrogen, Taastrup, Denmark) according to the manufacturer's instructions, and electroporated into electrocompetent *E. coli *TOP10 cells (Invitrogen) with a single pulse (2500 V, 400Ω, 25 μF) by use of a Gene Pulser apparatus (Bio-Rad Laboratories, Richmond, California). Plasmid DNA was isolated from the cells using the Qiagen Mini Spin Prep kit (QIAGEN), and subjected to PCR (HDA1/2-GC) as earlier described. The PCR products were run on a DGGE gel to check the purity and confirm the melting behavior of the excised band. The inserts were sequenced by GATC (Konstanz, Germany) using primers T3 and T7. The obtained sequences were compared to known sequences in the Ribosomal Database (RDP, Michigan State University, Release 9.61), and aligned using BLAST (bl2seq) and the GenBank database.

### Real-time PCR assay conditions

Real-time PCR was performed on samples from Experiment C on an ABI Prism 7900 HT from Applied Biosystems. The amplification reactions were carried out in a total volume of 20 μl containing 10 μl (2× PerfeCTA™ SYBR^® ^Green SuperMix, ROX from Invitrogen, Copenhagen, Denmark), primers (each at 200 nM concentration), 2 μl template DNA, and USB-H_2_O (USB EUROPE CMBH Staufen, Germany) purified for PCR. The amplification program consisted of one cycle at 50°C for 2 min; one cycle at 95°C for 10 min; 40 cycles at 95°C for 15 sec and 60°C for 1 min; and finally one cycle of melting curve analysis for amplicon specificity at 95°C for 15 sec, 60°C for 20 sec and increasing ramp rate by 2% until 95° for 15 sec. This program was found by preliminary experiments on target DNA in order to optimize reaction parametres and primer concentrations. The program was efficient and consistent for all primers used as seen by the high PCR efficiencies and correlation coefficients found (Table 6). The amplification products were further subjected to gel electrophoresis in 2% agarose, followed by ethidium bromide staining to verify amplicon sizes.

**Table 6:** Primers used for Real-Time PCR

Target gene	Forward primer (5'-3')	Reverse primer (5'-3')	Product size (bp)	PCR Efficiency (%)	Correlation coefficient (R^2^)	Reference
*Clostridium coccoides *16S	aaa tga cgg tac ctg act aa	ctt tga gtt tca ttc ttg cga a	440	97,8	0,998	[43]

*Bifidobacterium *16S	cgc gtc ygg tgt gaa ag	ccc cac atc cag cat cca	244	93,0	0,995	[44]

*Lactobacillus *16S	agcagtagggaatcttcca	caccgctacacatggag	341	98,6	0,998	[45,46]

*Bacteroides *spp.16S^a^	cgg cga aag tcg gac taa ta	acg gag tta gcc gat gct ta	360	100,1	0,997	This study

Butyryl-Coenzyme A	gcn gan cat ttc acn tgg aay wsn tgg cay atg	cct gcc ttt gca atr tcn acr aan gc	530	97,5	0,965	[21]

V2-V3 16S region (HDA)^b^	act cct acg gga ggc agc agt	gta tta ccg cgg ctg ctg gca c	200	113,7	0,991	[40]

^a^The bacteroides primer set was designed to amplify a segment of the DNA sequence represented by the highly homologous bands 4-7 in Table 5. ^b^PCR for the HDA primer set was run in parallel for each set of primers for all samples.

The *Bacteroides *spp. primer set was designed to amplify a segment of the DNA sequence represented by the highly homologous bands 4-7 in Table 3. ClustalW2 http://www.ebi.ac.uk/Tools/clustalw2/index.html was used to align these 4 sequences and NCBI's primer designing tool http://www.ncbi.nlm.nih.gov/tools/primer-blast/ was used to construct the primer set. Finally, the quality of the primer was checked with the Net Primer Software http://www.premierbiosoft.com/netprimer/index.html.

All results were calculated relatively as ratios of species DNA levels to HDA expression levels in order to correct data for differences in total DNA concentration between individual samples. DNA levels were approximated as 2^-Ct^, where C_t _is the threshold cycle calculated by the ABI software as the PCR cycle, where amplifications signal exceeds the selected threshold value, also set by the software. All samples were calculated as means of duplicate determinations. DNA isolation failed for one animal in the pectin group, hence the three experimental groups were: Control (N = 8), Apple (N = 8), and Pectin (N = 7).

### Statistics

Biomarker endpoints were tested for homogeneity of variance using Levene's test and for normal distribution by visual inspection of residual plots. Log-transformations were performed for data, which did not meet these criteria. The nonparametric Kruskal-Wallis test was used for datasets, which were not normally distributed or did not have homogeneity of variance even after log-transformation. Other data were after ANOVA analyzed by LSM (least square means). These statistical analyses were performed using the SAS Statistical Package, ver. 9.1.3 (SAS Institute Inc., Cary, NC). Statistical analysis of RT-PCR data was performed with SAS JMP version 6.0.2. Data was analyzed by one-way ANOVA followed by a pair-wise multiple comparison of means (Student's t). The significance level was set to P = 0.05.

## Authors' contributions

TRL and AW conceived of, designed and coordinated the microbiological investigations, and drafted the paper. MP and LOD conceived of, designed and coordinated the animal experiments. MH carried out the SCFA analyses, BNK carried out the enzyme analyses, AB carried out the RT-PCR measurements, and JM produced and characterized the apple products.
